# Supplementary material for: Mechanism of silica–lysozyme composite formation unravelled by in situ fast SAXS
Source: Beilstein J Nanotechnol. 2019 Jan 14;10:182–97. doi: 10.3762/bjnano.10.17 (PMC6350881; doi:10.3762/bjnano.10.17)
Supplement: File 1 — Additional experimental data. [file Beilstein_J_Nanotechnol-10-182-s001.pdf]

BEILSTEIN JOURNAL OF NANOTECHNOLOGY

Supporting Information

for

Mechanism of silica–lysozyme composite formation unravelled by in situ fast SAXS

Tomasz M. Stawski, Daniela B. van den Heuvel, Rogier Besselink, Dominique J. Tobler and Liane G. Benning

Beilstein J. Nanotechnol. **2019,** *10,* 182–197. doi:10.3762/bjnano.10.17

Additional experimental data

License and Terms: This is a supporting information file under the terms of the Creative Commons Attribution License (http://creativecommons.org/licenses/by/4.0). Please note that the reuse, redistribution and reproduction in particular requires that the authors and source are credited.
The license is subject to the *Beilstein Journal of Nanotechnology* terms and conditions: (https://www.beilstein-journals.org/bjnano)

Table S1: Selected physicochemical properties of the initial amorphous silica used in the experiments.

Molecular mass [g/mol]	60.08
Density [g/cm^3]	2.196
Scattering length density, SLD [$Å^{-2}$]	$1.883 \cdot 10^{-5}$
ΔSLD with respect to H_2O [$Å^{-2}$]	$9.362 \cdot 10^{-6}$
Solid amount from a 1000 ppm a sodium metasilicate solution at pH 7.5 and *T* = 21 °C [mmol/L]	14.85 mmol/L, equivalent to 0.041% volume fraction

Table S2: Silica size distribution from fitting.

Mean radius [nm]	2.525 ± 0.011
Variance [nm^2]	3.707 ± 0.029
Skew	3.976 ± 0.081
Kurtosis	24.80 ± 1.21

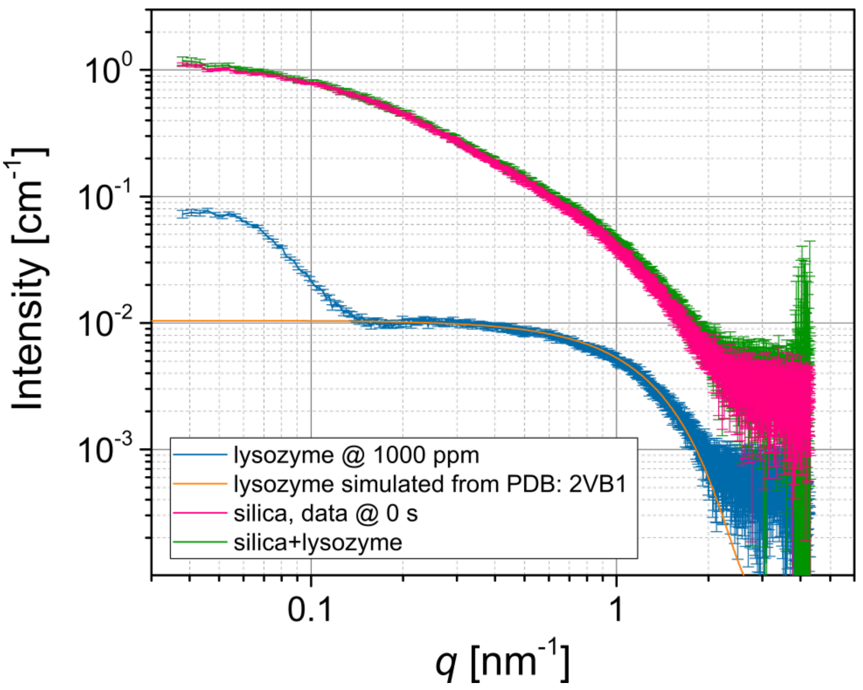

Figure S1: Scattering curves of solutions with the initial amorphous silica NPs and the LZM solutions measured independently prior to mixing. The pattern for lysozyme at 1000 ppm (the blue curve) matches well the simulated scattering pattern (the orange curve) generated from the PDB structure file 2VB1, with the exception of $q < 0.15$ nm^{-1}. The observed low-q intensity increase in the LZM solution originates from a very small population of larger particles/clusters/aggregates and is negligible. In the considered silica–LZM composites, the scattering contrast originating from the lysozyme itself can be mostly disregarded. This can be demonstrated by simply adding together the scattering intensities from the initial silica NP solution @ 0 s (the pink curve) and the LZM solution (the blue curve) and accounting for the uncertainties. The resulting pattern (the green curve) is within the uncertainty indistinguishable from the scattering of the silica NPs solution on its own. This addition corresponds to the hypothetical case when there were no interactions between the silica NPs and the protein, but it also points out that despite relatively high protein concentration (with respect to silica) the resulting scattering intensity of the protein is very low (see also [16] in the main text).
